# Correction: Dodd et al. Children’s Play and Independent Mobility in 2020: Results from the British Children’s Play Survey. *Int. J. Environ. Hum. Health* 2021, *18*, 4334

**DOI:** 10.3390/ijerph19159045

**Published:** 2022-07-25

**Authors:** Helen F. Dodd, Lily FitzGibbon, Brooke E. Watson, Rachel J. Nesbit

**Affiliations:** School of Psychology and Clinical Language Sciences, University of Reading, Reading RG6 6ES, UK; l.t.fitzgibbon@reading.ac.uk (L.F.); brooke.watson@pgr.reading.ac.uk (B.E.W.); r.j.nesbit@reading.ac.uk (R.J.N.)

**Table Legend**

In the original publication [1], there was a mistake in the legend for Table 6. The title was incorrect and should instead read “Geographic predictors of age children allowed out alone (independent mobility)”. The correct legend appears below.

**Table 6.** Geographic predictors of age children allowed out alone (independent mobility).

**Error in Figure/Table**

In the original publication, there was a mistake in Figure 1 and Tables 2–4 as published. The values calculated for hours spent playing, hours spent playing outside and hours spent playing adventurously were slightly miscalculated due to a minor error in the analysis code. This leads to slight changes in the values reported in the figure and tables.

The corrected Figure 1 and Table 2, Table 3 and Table 4 appear below.

**Figure 1:**
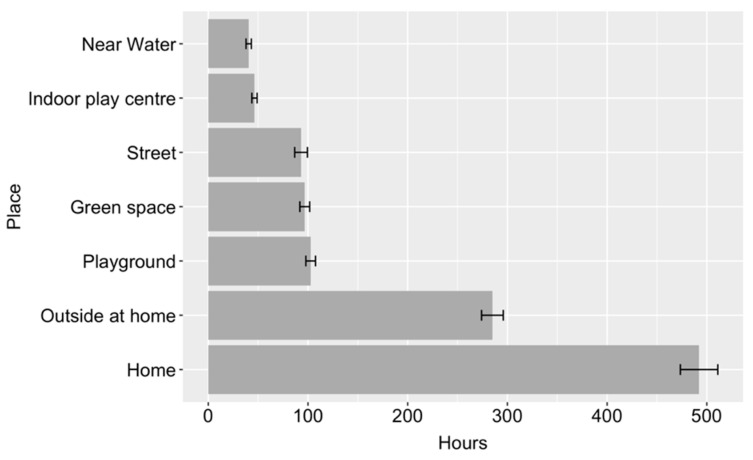
Mean time spent playing per year at each place (error bars show two standard errors).

**Table 2:** Socio-demographic predictors of hours spent playing, hours spent playing outside and hours spent playing adventurously per year.

	Hours Playing *p*/yr			Hours Playing Outside *p*/yr			Hours Playing Adventurously *p*/yr		
Predictors	Estimates	CI	*p*	Estimates	CI	*p*	Estimates	CI	*p*
(Intercept)	32.75	30.91–34.59	**<0.001**	23.90	22.26–25.54	**<0.001**	27.56	25.06–30.07	**<0.001**
Child age	−0.58	−0.86–−0.30	**<0.001**	−0.16	−0.41–0.08	0.192	−0.74	−1.13–−0.35	**<0.001**
Child sex: Male	Reference			Reference			Reference		
Child sex: Female	−1.21	−2.24–−0.17	**0.022**	−1.06	−1.97–−0.15	**0.022**	−2.23	−3.69–−0.78	**0.003**
Parent social grade: ABC1	Reference			Reference			Reference		
Parent social grade C2DE	1.28	0.15–2.42	**0.026**	0.47	−0.55–1.49	0.367	0.17	−1.42–1.76	0.834
Child disability: No	Reference			Reference			Reference		
Child disability: Yes	−1.16	−2.77–0.44	0.155	−1.61	−3.02–−0.20	**0.025**	−3.55	−5.91–−1.19	**0.003**
Parent health/disability: No	Reference			Reference			Reference		
Parent health/disability: Yes, limited a lot	2.15	0.23–4.06	**0.028**	3.72	1.93–5.51	**<0.001**	4.15	1.54–6.76	**0.002**
Parent health/disability: Yes, limited a little	0.84	−0.75–2.42	0.301	1.15	−0.30–2.59	0.120	1.28	−0.94–3.50	0.257
Parent ethnicity: White	Reference			Reference			Reference		
Parent ethnicity: Minority	−2.12	−3.57–−0.67	**0.004**	−0.58	−1.89–0.72	0.379	−1.32	−3.41–0.77	0.216
Employment: Full time	Reference			Reference			Reference		
Employment: Part time	2.27	1.01–3.53	**<0.001**	2.01	0.89–3.12	**<0.001**	2.34	0.56–4.12	**0.010**
Employment: Unemployed/other	2.12	0.69–3.55	**0.004**	0.95	−0.32–2.23	0.143	1.78	−0.24–3.80	0.084
Birth order: First born	Reference			Reference			Reference		
Birth order: Not first born	0.06	−1.10–1.22	0.918	0.44	−0.58–1.45	0.401	−0.90	−2.52–0.73	0.280
Parent age: Younger	Reference			Reference			Reference		
Parent age: Middle	−1.95	−3.42–−0.49	**0.009**	−1.49	−2.79–−0.19	**0.025**	−1.78	−3.84–0.27	0.089
Parent age: Older	−4.13	−5.76–−2.49	**<0.001**	−3.21	−4.70–−1.72	**<0.001**	−3.74	−6.03–−1.46	**0.001**
Parent education: Low	Reference			Reference			Reference		
Parent education: Med	−0.15	−1.53–1.22	0.826	−0.65	−1.87–0.56	0.294	0.38	−1.52–2.28	0.692
Parent education: High	0.95	−0.47–2.36	0.192	−0.69	−1.95–0.58	0.286	0.65	−1.37–2.68	0.526
Observations	1346			1346			1263		
R^2^/R^2^ adjusted	0.081/0.072			0.059/0.049			0.063/0.053		

Note. Bold *p*-values are <0.05, indicating statistical significance.

**Table 3:** Geographic predictors of hours spent playing, hours spent playing outside and hours spent playing adventurously per year.

	Hours Playing *p*/yr			Hours Playing Outside *p*/yr			Hours Playing Adventurously *p*/yr		
Predictors	Estimates	CI	*p*	Estimates	CI	*p*	Estimates	CI	*p*
(Intercept)	33.78	32.23–35.32	**<0.001**	25.35	23.97–26.72	**<0.001**	27.43	25.15–29.72	**<0.001**
Region: Scotland	Reference			Reference			Reference		
Region: London	−1.43	−3.19–0.33	0.112	−2.47	−4.03–−0.90	**0.002**	−1.24	−3.82–1.34	0.348
Region: North	−1.70	−3.52–0.12	0.067	−3.07	−4.67–−1.48	**<0.001**	−2.08	−4.79–0.62	0.131
Region: Midlands	−1.67	−3.69–0.35	0.106	−2.81	−4.56–−1.06	**0.002**	−2.34	−5.28–0.59	0.118
Region: East	−3.69	−5.74–−1.64	**<0.001**	−4.38	−6.23–−2.53	**<0.001**	−2.31	−5.30–0.68	0.130
Region: South	−0.81	−2.57–0.94	0.365	−2.18	−3.72–−0.64	0.006	−0.48	−3.05–2.09	0.717
Region: Wales	−1.72	−4.49–1.05	0.224	−2.32	−4.74–0.10	0.060	−1.51	−5.29–2.26	0.432
Location: Urban	Reference			Reference			Reference		
Location: Town and fringe	−0.33	−1.82–1.16	0.663	0.71	−0.56–1.99	0.272	0.20	−1.91–2.31	0.853
Location: Rural	0.03	−1.45–1.51	0.969	0.81	−0.48–2.10	0.218	1.01	−0.86–2.88	0.291
Observations	1919			1919			1788		
R^2^/R^2^ adjusted	0.008/0.004			0.016/0.012			0.004/−0.000		

Note. Bold *p*-values are <0.004, indicating Bonferroni-corrected statistical significance.

**Table 4:** Parent attitude towards risk predictors of hours spent playing, hours spent playing outside and hours spent playing adventurously per year.

	Hours Playing *p*/yr			Hours Playing Outside *p*/yr			Hours Playing Adventurously *p*/yr		
Predictors	Estimates	CI	*p*	Estimates	CI	*p*	Estimates	CI	*p*
(Intercept)	32.27	31.83–32.72	**<0.001**	23.00	22.62–23.38	**<0.001**	26.20	25.58–26.83	**<0.001**
Engagement with risk	1.26	0.78–1.74	**<0.001**	0.81	0.38–1.24	**<0.001**	2.02	1.34–2.71	**<0.001**
Protection from injury	0.14	−0.35–0.64	0.577	−0.23	−0.65–0.19	0.274	−0.34	−1.07–0.38	0.355
TRiPs	0.87	0.33–1.40	**0.001**	1.21	0.76–1.67	**<0.001**	0.97	0.21–1.72	**0.013**
Observations	1919			1919			1788		
R^2^/R^2^ adjusted	0.030/0.029			0.042/0.040			0.037/0.035		

Note. Bold *p*-values are <0.05, indicating statistical significance.

**Text Correction**

There was an error in the original publication. As described above, the values calculated for hours spent playing, hours spent playing outside and hours spent playing adventurously were slightly miscalculated due to a minor error in the analysis code. Given this, a number of corrections have been made.

A correction has been made to **Results**, **Section 3.1**, **Paragraphs 1, 2 and 3**:

To address research question 1, three variables were used from the CPS: total time spent playing across the year, total time spent playing outside across the year, and total amount of time spent playing adventurously across the year. As expected, given there is an overlap in the items used to create the scores, these three measures from the CPS were all significantly correlated (*rs* ≥ 0.70, *p* < 0.001).

Children were reported to spend an average of 1140 h (*SD* = 641 h) playing per year. Of that time, 604 h (*SD* = 403 h), or 53%, was spent playing outside, and 133 h (*SD* = 133 h), or 12% of their total play time, was spent playing in nature.

Figure 1 shows the mean number of hours that children were reported to spend playing at each of the provided locations, across a year. The average total time children spent playing varied significantly across place, *F* (6,1912) = 958.37, *p* < 0.001. Coefficients demonstrated significant differences (at Bonferroni corrected alpha value of 0.002) between all places. Children spent the most time playing at home or at other people’s homes and the least time playing near water and at indoor play facilities, including swimming pools, trampoline parks and soft play. Away from home, children on average spent more time playing at playgrounds than in any other place.

A correction has been made to **Results**, **3.4.1. Socio-Demographic Factors**, **Paragraphs 2, 3 and 4**:

For total hours spent playing, the results indicate that child age, child sex, social grade, ethnicity, full time employment status (relative to working part time and not working/other) and respondent age were significant predictors. Parent disability status was not a significant predictor after Bonferroni correction for multiple comparisons. The children who played the most were younger and male, and their responding parent/caregiver was of lower social class, white, did not work full time and was relatively young in comparison to other respondents. 

For hours spent playing outdoors, child sex, child disability, respondent health problem/disability, respondent full time employment status (relative to working part time) and respondent age were significant predictors of children’s time spent playing outdoors. The children who played outdoors the most were males who did not have a disability and whose responding caregiver was relatively young and worked part-time. Perhaps surprisingly, children whose responding parent/caregiver had a health condition or disability that significantly limited them spending more time playing outdoors than those whose parents were healthy or only limited a little by health or disability.

For time spent playing adventurously, child sex, child age, child disability, respondent health problem/disability, respondent full time employment status (relative to working part time) and respondent age were all significant predictors. The children who spent the most amount of time playing adventurously were boys, younger children, children who did not have a disability themselves and children whose responding parent/caregiver was white and working part-time. As with outdoor play, having a responding parent/caregiver with a limiting disability or health condition was related to more time spent playing adventurously. 

A correction has been made to **Results**, **3.4.2. Geographic Factors**, **Paragraph 2**:

A significant main effect was found for region. To reduce the number of comparisons, we used Scotland, which had the highest play hours, and the East of England, which had the lowest play hours, as the reference categories, although results are only presented in the tables for Scotland as the reference to reduce the size of tables. Bonferroni corrected alpha of 0.004 was applied to these comparisons. The coefficients in Table 3 show that, relative to children in Scotland, children in the East of England spent significantly less time playing. Relative to children in the East of England, children in the South of England, and Scotland spent more time playing. For time playing outdoors, children in London, the North of England, the Midlands, and the East of England spent less time playing outdoors than children in Scotland. Relative to children in the East of England, children in the South of England, and Scotland spent more time playing outdoors. Time spent playing adventurously did not differ significantly across regions. For all three play variables, there were no significant differences between children living in an urban area and children living either in town/fringe areas or rural areas. It is important to note that, although these differences across regions are statistically significant, the proportion of variance accounted for by geographical locations overall is consistently less than 2%, indicating that regional differences are very small.

A correction has been made to **Discussion**, **4.1**, **Paragraph 1**:

The first research question focused on where children spend time playing. Our results showed that children spent on average 1140 h a year playing, which equates to an average of 3.12 h per day, although there is considerable variation between children. Consistent with previous research [9] and unsurprisingly, the place where children played the most was indoors at home or in other people’s homes. Outdoor play accounted for around half of children’s play and most commonly happened in gardens at home or in other people’s gardens. This is also consistent with previous research from Norway, showing that gardens are the most common outdoor space used for play [12]. Away from home, playgrounds were the most common place for children to play, followed by green spaces such as forests and grassy spaces and then on the street and local public spaces. This highlights the importance of public play spaces, such as playgrounds and green spaces, especially for those children who do not have access to a garden at home.

A correction has been made to **Discussion**, **4.4**, **Paragraph 1**:

The final two research questions focused on how geographic location, socio-demographic factors and parent/caregiver attitudes were related to children’s play and independent mobility. An important starting point for discussing these findings is to highlight that none of these predictors accounted for a large amount of variance in children’s play or independent mobility. Geographic factors explained very little variance in children’s play (<2%) but were more important for independent mobility, explaining 5% of variance. In contrast, socio-demographic factors were the strongest predictor of children’s play, accounting for around 5–7% of variance but explained less than 1% of variance in independent mobility. Parent attitudes were the strongest predictor for independent mobility, accounting for around 9% of variance in the age that children were allowed out alone. They accounted for between 3–4% of variance in play measures, being a stronger predictor of adventurous and outdoor play than total play. This is perhaps not surprising given that the measures focused on risk tolerance which we would expect to be linked to children’s risk taking during adventurous play.

A correction has been made to **Discussion**, **4.4**, **Paragraphs 4 and 5**:

For socio-demographic factors, a range of these were associated with children’s play and these differed by the type of play. Children played less and played less adventurously as they got older. Across all play variables, girls played less than boys, but this difference was largest for time spent playing adventurously. Children whose participating parent was from a lower social grade spent more time playing overall, but this effect was not found for outdoor or adventurous play, indicating that these children spend more time playing, but primarily at home or in other people’s homes. In contrast, child disability was only related to hours spent playing outside and adventurously; children reported to have a diagnosed learning difficulty, a mental health problem or a physical disability spent less time playing outdoors. Perhaps surprisingly, children whose responding parent/caregiver reported that they had a health problem or disability within the past 12 months played more across all measures than children whose responding parent did not have a health problem or disability. In general, children whose responding parent/caregiver was white played more than children with a non-white parent/caregiver, but only when looking across all play locations and not for outdoor or adventurous play, and children played more if their parent/caregiver worked part-time relative to full time and if their parent/caregiver was relatively young.

To our knowledge, only one study has previously examined predictors of children’s time spent outdoors in Britain [20]. In this study, correlations of time outdoors, rather than play specifically, were examined. Boys from a lower SES background who spent less than 2 h a day on a computer were found to spend more time outside. Our findings are only partially consistent with these; we found that children from lower SES backgrounds played more but SES was not a significant predictor of outdoor play. This inconsistency may be explained by our focus on play rather than time outdoors, our use of a nationally representative sample rather than a geographically limited opportunity sample, or the inclusion of other correlates of play within the same model that may explain some of the variance that might have been accounted for by SES. Our results are broadly consistent with previous international research. For example, Parent et al. [18] also found that play was associated with ethnicity and a recent review highlighted consistent associations between outdoor play and maternal employment status [19].

A correction has been made to **Conclusions**, **5**:

The results of the British Children’s Play Survey presented here show that on average, children living in Britain in 2020 play for just over 3 h per day. Around half of children’s play happens outdoors. Away from home, playgrounds and green spaces are the most common places for children to play. The most adventurous places for play were green spaces, indoor play centres, including soft play, trampoline parks and swimming pools, followed by playgrounds and near water. A significant difference was found between the age that children are now allowed out alone in comparison to the previous generation, with children now almost two years older than their parents/caregivers were when granted independent mobility. A range of socio-demographic factors predicted children’s play, with the most consistent findings found for child age, child sex, parent age and parent employment status, with younger children whose responding parent was younger and worked part-time, playing the most. There was little evidence that geographic location had a substantial impact on children’s play, but it was important for independent mobility, with children living in town/fringe areas and children living in Scotland allowed out alone at a younger age. When parents/caregivers had more positive attitudes around children’s risk-taking in play, children spent more time playing and were able to be out of the house independently at a younger age.

The authors apologize for any inconvenience caused and state that the scientific conclusions are unaffected. The original publication has also been updated.
